# Microbial Diversity in African Foods and Beverages: A Systematic Assessment

**DOI:** 10.1007/s00284-023-03481-z

**Published:** 2023-11-26

**Authors:** Soumya Ghosh, Charné Bornman, Maryam Meskini, Mehri Joghataei

**Affiliations:** 1 Department of Genetics, Faculty of Natural and Agricultural Sciences, University of the Free State, Bloemfontein, 9301 South Africa; 2 Department of Engineering Sciences, Faculty of Natural and Agricultural Sciences, University of the Free State, Bloemfontein, 9301 South Africa; 3 Microbiology Research Centre, Pasteur Institute of Iran, Teheran, Iran; 4 Mycobacteriology & Pulmonary Research Department, Pasteur Institute of Iran, Teheran, Iran; 5 Student Research Committee, Pasteur Institute of Iran, Tehran, Iran; 6 Department of Food Science and Technology, Faculty of Agriculture, Ferdowsi University of Mashhad, Mashhad, Iran

## Abstract

This article provides a comprehensive and in-depth examination of the microbial diversity inherent in African food and beverages, with a particular emphasis on fermented products. It identifies and characterizes the dominant microorganisms, including both prokaryotes and yeasts, prevalent in these foods, and furthermore, critically analyzes the health benefits of these microbial strains, especially their probiotic properties, which could potentially improve digestion and contribute to human health. Notably, it underscores the vital role these microorganisms play in bolstering food security across Africa by enhancing and preserving food quality and safety. It also delves into the potential applications of microbial products, such as metabolites, in the food industry, suggesting their possible use in food processing and preservation. Conclusively, with a summarization of the key findings, emphasizing the importance of gaining a deep understanding of microbial diversity in African beverages and foods. Such knowledge is crucial not only in promoting food security but also in advancing public health.

**Graphical Abstract:**

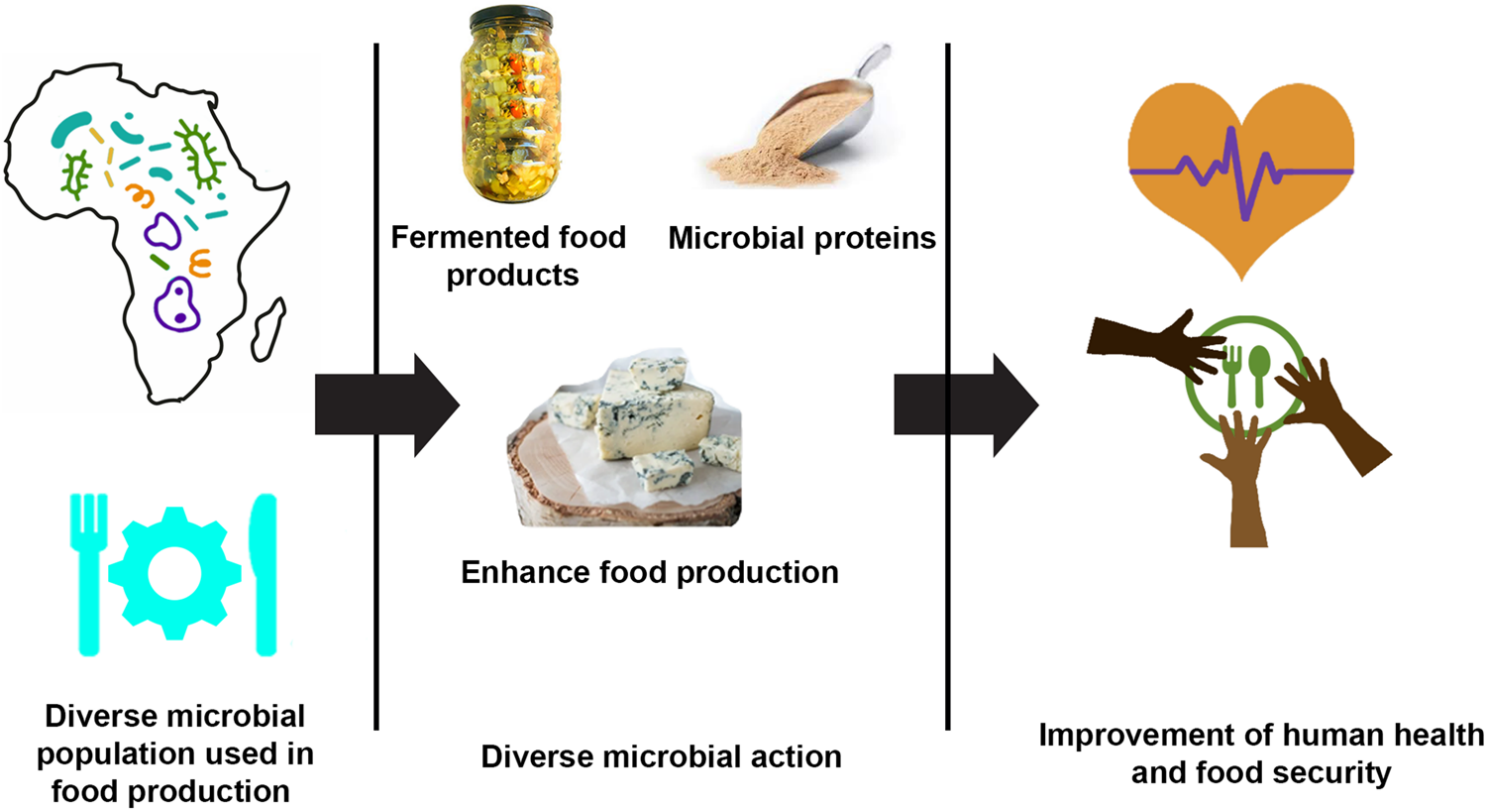

## Introduction

This article is an exploration of the pivotal role microbial diversity plays in food security and human health on the African continent. Boasting a diverse array of microorganisms, Africa's rich reservoir serves numerous functions, including food preservation, nutritional enhancement, boosting food production, and even acting as a food source itself [1, 2].

A significant aspect of the African diet is the age-old tradition of consuming fermented foods [2]. This process, driven by microorganisms that convert carbohydrates into organic acids, alcohols, or other easily digestible products, not only enriches the food's flavor but also enhances nutritional value and extends shelf life [3]. Fermented foods in Africa are largely grouped into non-alcoholic fermented cereals, starchy root crops, vegetable proteins, animal proteins, and alcoholic beverages. Each of these fermented food types plays a pivotal role in the African diet due to the wide range of substances that can be fermented [4]. Most fermented foods and beverages worldwide, including those in Africa, are naturally fermented by a combination of cultivable and non-cultivable microorganisms, primarily sourced from raw materials and processing facilities [5].

In addition, the study of the microbial ecology of natural food fermentations can assist in identifying biomarkers for assessing fermented food quality and aid in the development of optimal starter cultures [6]. Microbial ecology is the study of microorganisms in their natural environments [7]. In the context of food, this involves the study of the various bacteria, yeasts, and molds that play a role in the fermentation process. Identifying biomarkers, which are measurable indicators of some biological state or condition, can help assess the quality of fermented foods. For example, certain microbial metabolites, enzymes, and proteins can be used as biomarkers to determine whether a food product has been properly fermented or if it contains harmful microorganisms [8].

Furthermore, the article investigates the probiotic properties of the dominant strains of microorganisms found in African foods and beverages. Probiotics, live microorganisms that confer health benefits to the host when consumed in adequate amounts, present a vast potential for new probiotic strain discovery due to Africa's microbial diversity. These strains could provide numerous health benefits, including improved digestion, enhanced immunity, and potential protection against certain diseases [9]. This exploration holds significant potential for both the health of the African population and global health applications.

This article also explores deeper into the significance of microbial proteins in the food industry, their role in producing a wide array of products, and their contribution to food security. Finally, the critical role microorganisms play in ensuring food securty is emphasized. The increasing global population and the subsequent demand for food necessitate sustainable food production methods. Microorganisms can play a crucial role in addressing food security issues. This is particularly relevant in Africa, where food insecurity is a pressing issue. Efficient utilization of microbial resources could significantly contribute to sustainable food production and security. This paper aims to illuminate these aspects, revealing the potential of microbial diversity in Africa's food and beverage industry. This article concludes by highlighting the existing knowledge gaps and potential areas for future research.

## Microorganisms Commonly Used in African Foods and Beverages and Their Probiotic Properties

Traditional fermented foods play an important part in African food regime, as far as fermentation process facilitates food preservation, increases the shelf life, and promotes the nutritional value of the food products. One the most important aspects of any fermentation process is the associated food microbiota and the studies on African fermented foods and beverages have revealed the presence of diverse microbial populations in which most of them have technological, fundamental commercial importance [2, 10, 11]. According to these indicated studies, African fermented foods are categorized as non-alcoholic fermented cereals (millet, sorghum, and maize), starchy root crops (cassava), vegetable proteins (oilseeds and legumes), animal proteins (dairy), and alcoholic beverages (sap, fruits, honey, or cereals). The microbial consortium is multifaceted and consists of numerous distinct microorganisms that coexist and interact in different ways. Some organisms are killed or inactivated during the fermentation process, while strains better suited to fermentation are activated and undergo rapid growth [12]. The entire competitive microbial activities result in spontaneous fermentation. During fermentation, the lactic acid bacteria that survives make a synergistic correlation with some yeasts. Table 1 lists the microbial flora of drinks and fermented food in Africa, the continent of original and natural substrates.

**Table 1:** Dominant microorganisms in African foods

Continental Zones	Country	Food Products	Fermentation substrates	Fermentation microorganisms (indigenous / deliberate inoculation)	References
Northern Africa	Sudan	Kisra	Sorghum	LAB	[51]
		Hussuwa	Sorghum	*L*. *fermentum*, *Ped*. *acidilactici*, *Ent. faecium (minor proportions)*	[52]
		Merissa (alcoholic beverage)	Sorghum	Lactic acid bacteria, acetic acid bacteria	[53]
	Morocco	Leban (sour milk)	Milk	*Lactic streptococci (lactococci)*, *Leuconostoc lactis*, *Leuconostoc mesenteroides* subsp. *cremoris*	[52]
	Egypt	Bouza (alcoholic beverage)	Wheat or maize	Unknown	[53]
		Zabady	Buffalo and cow milk	*Lactococcus garvieae*, *Streptococcus thermophilus*, *Lactococcus raffinolactis*, *Leuc*. *citreum*, *Lc*. *lactis*, *Lb*. *johnsonii*, *Lc*. *delbrueckii* subsp. *bulgaricus*	[54, 55]
		Borde	Barley	*Lactobacillus* spp.	[56]
		Kishk	Yoghurt and bulgur	*B*. *licheniformis*, *B*. *subtilis*, and *B*. *megatherium*, Lactic acid bacteria	[57]
	Algeria	Leben (lben)	Goat, sheep or cow Milk	*S*. *cerevisiae*, *Kluyveromyces lactis*, *Leuc*. *mesenteroides* subsp. *dextranicum*, *Lc*. *lactis* subsp. *cremoris*, *Leuc*. *lactis*, *E*. *faecalis*, *E*. *faecium*, *S*. *thermophilus*, *Lc*. *lactis*, *Lc*. *lactis* subsp. *lactis*, *E*. *faecalis*	[58]
	North Africa	Leben (lben)	Goat, sheep or cow Milk	*S*. *cerevisiae*, *Kluyveromyces lactis*, *Leuc*. *mesenteroides* subsp. *dextranicum*, *Lc*. *lactis* subsp. *lactis biovar diacetylactis*, *Lb*. *brevis*, *Lc. lactis* subsp. *cremoris*, *Leuc*. *lactis*, *E*. *faecalis*, *E*. *faecium*, *S*. *thermophilus*, *Lc*. *lactis*, *Lc*. *lactis* subsp. *lactis*, *E*. *faecalis*	[58, 59]
Eastern Africa	Kenya	Uji	Maize, sorghum, or millet	*L*. *plantarum*, *L*. *paracasei*, *L*. *fermentum*, *L*. *buchneri*, *Pediococcus acidilactici*, *P*. *pentosaceus*	[60]
		Busaa (alcoholic beverage)	Maize	Yeasts *and Lactobacillus* spp.	[53]
		Kule-naoto	Milk	*L*. *plantarum*, *L*. *fermentum*, *L*. *paracasei*, *L*. *acidophilus*, *Lactococci*, *Leuconostocs*, and *Enterococci*	[52]
		Maziwalala	Milk	*“Strep.” (Lact.) lactis*, *Strep*. *thermophilus*	[52]
		Bushera	Sorghum or millet	*Lactobacillus* spp., *Streptococcus* spp., *Leuconostoc* spp., *Pediococcus* spp., *Weissella* spp.	[61]
		Suusac	Camel Milk	*C*. *inconspicua*, *Cryptococcus laurentii*, *C*. *famata*, *C*. *lusitaniae*, *S*. *cerevisiae*, *Trichosporon mucoides*, *Rhodotorula mucilaginosa*, *Trichosporon cutaneum*, *Geotrichum penicillatum*, *C*. *krusei*, *Leuc*. *mesenteroides* subsp. *mesenteroides*, *Lb*. *helveticus*, *W*. *confusa*, *E*. *faecium*, *S*. *salivarius/thermophilus*, *E*. *faecalis*, *Lc*. *Lactis* subsp. *lactis*, *Lb*. *fermentum*, *Leuc*. *lactis*, *Lb*. *curvatus*, *Lb*. *plantarum*, *Leuc*. *mesenteroides*, *Lc*. *raffinolactis*, *Lb*. *salivarius*	[41]
		Amabere	Cow Milk	*Trichosporon mucoides*, *S*. *cerevisiae*, *C*. *albicans*, *C*. *famata*, *Lb*. *fermentum*, *Lb*. *helveticus*, *Lb*. *plantarum*, *S*. *thermophilus*, *Leuc*. *mesenteroides*, *Lb*. *bulgaricus*	[45, 62]
		Kule-naoto	Cow Milk	*Leuc*. *mesenteroides*, *Lb*. *acidophilus*, *Lc*. *lactis*, *Lb*. *fermentum*, *E*. *faecium*, *Lb*. *plantarum*, *Lb*. *casei*, *Lb*. *paracasei*, *Lb*. *rhamnosus*	[63, 64]
		Mursik	Cow or goat Milk	*S*. *fermentati*, *C*. *sphaerica*, *C*. *kefyr*, *C*. *krusei*, *Lb*. *pontis*, *Lb*. *brevis*, *Lb*. *rhamnosus*, *Lb*. *casei*, *Lb*. *kefiri*, *Lb*. *paracasei*, *Cereus*, *Lb*. *helveticus*	[65, 66]
	Malawi	Nsima	Fermented maize porridge	Unknown	[67]
	Ethiopia	Injera	Sorghum	*Candida guillermondii*	[52]
		Kocho	Ensette or Abyssinian banana (Ensette ventricosum)	LAB, yeast	[52]
		Ergo	Cow Milk	*Lc*. *lactis* subsp. *cremoris*, *Lb*. *mesenteroides*, *Lc*. *lactis* subsp. *lactis*, *S*. *thermophilus*, *Leuc*. *cremoris*, *Lb*. *delbrueckii*, *Micrococcus* spp., *Lb*. *homi*	[52] and [66, 68]
		Tej (alcoholic beverage)	Honey	Yeasts	[69]
		Teff dough	Teff	*L*. *brevis*, *L*. *fermentum*, *L*. *plantarum*, and/or *Pediococcus pentosaceus*, *L*. *sanfranciscensis*	[70]
		Obushera	Millet	LAB	[52]
		Tonton	Banana	Unknown	[2]
		Malawa beer (alcoholic beverage)		*Candida krusei*	[52]
		Togwa	Cassava, maize, sorghum, millet	*Lactobacillus* spp., *Pediococcus pentosaceus*, *Weissella* *confusa*, *Issatchenkia orientalis*, *S*. *cerevisiae*, *Candida pelliculosa*, and *C*. *tropicalis*	[71]
		Ititu	Cow Milk	*Lb*. *plantarum*, *Lb*. *casei*	[72, 73]
	Tanzania	Kivunde	Cassava	*L. plantarum*, *other LAB*, *yeast*	[52]
		Cingwada	Cassava	Unknown	[52]
	Zambia	Zambian opaque maize beer (alcoholic beverage)	Maize	Yeasts	[52]
	Sudan	Rob	Goat, sheep or cow Milk	*C*. *kefyr*, *S*. *cerevisiae*, *Lb*. *acidophilus*, *Lc*. *lactis*, *Lb*. *fermentum*, *Strep*. *salivarius*	[74]
	Somalia	Suusac	Camel Milk	*C*. *inconspicua*, *Cryptococcus laurentii*, *C*. *famata*, *C*. *lusitaniae*, *S*. *cerevisiae*, *Trichosporon mucoides*, *Rhodotorula mucilaginosa*, *Trichosporon cutaneum*, *Geotrichum penicillatum*, *C*. *krusei*, *Leuc*. *mesenteroides* subsp. *mesenteroides*, *Lb*. *helveticus*, *W*. *confusa*, *E*. *faecium*, *S*. *salivarius/thermophilus*, *E*. *faecalis*, *Lc*. *Lactis* subsp. *lactis*, *Lb*. *fermentum*, *Leuc*. *lactis*, *Lb*. *curvatus*, *Lb*. *plantarum*, *Leuc*. *mesenteroides*, *Lc*. *raffinolactis*, *Lb*. *salivarius*	[41, 75]
	Uganda	Kwerionik	Cow Milk	*Leuc*. *mesenteroides* subsp. *mesenteroides*, *Lb*. *casei* subsp. *casei*, *E*. *faecalis*, *Lb*. *plantarum*, *Lb*. *paracasei* subsp. *paracasei*, *E*. *faecium*, *Lc*. *lactis* subsp. *lactis*	[72, 76]
		Makamo	Milk	ND	[77]
Western Africa	Mozambique	Masse	Cow Milk	*Leuc*. *garlicum*, *Leuc*. *pseudomesenteroides*, *Lc*. *lactis* subsp. *lactis*, *Leuc*. *lactis*	[77, 78]
	Northern Ghana	Nunu	Cow Milk	*C*. *rugosa*, *S*. *cerevisiae*, *C*. *tropicalis*, *Pichia kudriavzevii*, *C*. *parapsilosis*, *Galactomyces geotrichum, E*. *italicus*, *Lactococcus* spp., *Lb*. *plantarum*, *Lb*. *fermentum*, *Leuc*. *mesenteroides*, *E*. *faecium*, *Lb*. *helveticus*, *W*. *confusa*	[40, 79]
	Southern Ghana	Nyarmie	Cow Milk	*S*. *cerevisiae*, *Trichosporon cutaneum*, *Candida* spp., *Lc*. *lactis*, *S*. *thermophilus*, *Lb*. *helveticus*, *Lb*. *delbrueckii* subsp. *lactis*, *Leuc*. *mesenteroides* subsp. *mesenteroides*, *Lb*. *delbrueckii* subsp. *bulgaricus*	[48, 78]
	Namibia	Omashikawa	Not specified Milk	*Lb*. *helveticus*, *Lb*. *casei*, *Lb*. *paracasei*, *Lc*. *lactis* subsp. *lactis*, *Lb*. *rhamnosus*, *E*. *faecium*, *Leuc*. *pseudomesenteroides*, *E*. *durans*, *Lb*. *kefiri*	[66, 77]
	Cameroon	Pendidaam	Not specified Milk	*E*. *faecium*, *Leuc*. *mesenteroides*, *S*. *thermophilusc*, *Lb*. *fermentum*, *Streptococcus*, *Lb*. *delbrueckii*, *Enterococcus* spp., *Lb*. *helveticus*, *Lb*. *plantarum*, *E*. *faecalis*, *Lb*. *casei*, *Leuc*. *paramesenteroides*	[80, 81]
		Kindirmou	Not specified Milk	*ND*	[66, 82]
	Mali	Fene	Milk	*W*. *confusa*, *Lb*. *fermentum*, *Enterococcus* spp., *Lc*. *lactis* subsp. *lactis*, *Lb*. *plantarum*, *P*. *pentosaceus*	[78, 83]
Middle Africa	Congo	Potopoto	Maize	*L*. *gasseri*, *L*. *plantarum/paraplantarum*, *L*. *acidophilus*, *L*. *delbrueckii*, *L*. *reuteri*, *L*. *casei*, *Bacillus* spp., *Enterococcus* spp.	[52]
		Chikwangue	Cassava	LAB, yeast	[52]
		Koko and Kenkey	Maize, sorghum, or millet	*W*. *confusa*, *L*. *fermentum*, *L*. *salivarius*, *L*. *vaccinostercus*, *L*. *pantheris*, *Pediococcus* spp., and yeasts	Bonome (stink fish)
		Agbelima	cassava	*L*. *plantarum*, *L*. *brevis*, *L*. *fermentum*, *Leuc*. *mesenteroides*, also *Bacillus* spp., *Candida tropicalis*, *Geotrichum candidum*, *Penicillium* spp.	[84]
		Bonome (stink fish)	Fish	Unknown	[52]
		Fura	Millet	*Lb*. *fermentum*, *Lb*. *reuteri*, *W*. *confusa*, *Pd*. *acidilactici*	[85]
		Gari	Cassava	*Lactobacillus plantarum*, *Leuconostoc mesenteroides*, *Bacillus subtilis*, *Klebsiella* sp., *Candida tropicalis*, *C*. *krusei*	[86]
		Kokonte	Cassava	*Bacillus* spp., *Geotrichum* spp., and *Lactobacillus* spp.	[86]
		Nunu	Milk	*Lactobacillales*, *Enterobacteriales*, *Streptococcus infantarius*	[10]
		Burukutu(alcoholic beverage)	Sorghum	Yeasts, and lactic acid bacteria	[53]
		Pito (alcoholic beverage)	Sorghum	Molds, yeasts, and *Lactobacillus* spp.	[87]
	Benin	Mawe	Maize	*L*. *lactis*, *P*. *pentosaceus*, *L*. *plantarum*	[84]
		Dawadawa or iru	African locust bean (Parkia biglobosa) Soybean	*Bacillus subtilis*, *B*. *licheniformis*	[88]
		Gowe	Sorghum and/or maize flour	*L*. *fermentum*, *W*. *confusa*, *L*. *mucosae*, *P*. *acidilactici*, *P*. *pentosaceus*, *W*. *kimchii*, *K*. *marxianus*, *P*. *anomala*, *C*. *krusei*, *C*. *tropicalis*	[89]
		Kunu-zaki	Sorghum, Millet	*L*. *fermentum*, *P. pentosaceus*, *W*. *confusa*, *E*. *faecalis*	[32]
		Lafun	Cassava	*L*. *fermentum*, *L*. *plantarum*, *W*. *confusa*, yeasts (*Saccharomyces cerevisiae*, *Pichia* *scutulata*, *Klyveromyces marxianus*, *Hanseniaspora guillermondii*), and *Bacillus* spp.	[52]
		Fufu	Cassava	*P*. *pentosaceus*, *L*. *fermentum*, *L*. *plantarum*	[52]
		Ogi	Maize, sorghum, or millet	*P*. *pentosaceus*, *L*. *fermentum*, *L*. *plantarum*, yeast (*Saccharomyces cerevisiae*, *Candida kruseii*)	[90]
		Palm wine (alcoholic beverage)	Palm sap	Yeasts	[91]
		Ogiri	Melon (Citrullus vulgaris)	*Bacillus* sp. (*predominant*), *Proteus*, *Pediococcus*	[52]
		Ogiri-nwan	Fluted pumpkin bean (*Telfairia occidentalis*)	*Bacillus* sp. (*proteolytic*)	[91]
		Ogiri-igbo (ogiri-agbor)	Castor oil seed (*Ricinus communis)*	*Various Bacillus* sp.: *B*. *subtilis*, *B*. *megaterium*, *B*. *firmus*	[91]
		Ugba (apara)	African oil bean (Pentaclethra macrophylla)	*Bacillus subtilis*, *Micrococcus* sp.	[91]
		*Owoh*	Cotton seeds (*Gossypium hirsutum*)	*Bacillus* sp.	[92]
		Sheketeh (alcoholic beverage)	Maize	Unknown	[53]
		Nunu	Cow milk	*C*. *rugosa*, *S*. *cerevisiae*, *C*. *tropicalis*, *Pichia kudriavzevii*, *C*. *parapsilosis*, *Galactomyces geotrichum*, *E*. *italicus*, *Lactococcus* spp., *Lb*. *plantarum*, *Lb*. *fermentum*, *Leuc*. *mesenteroides*, *E*. *faecium*, *Lb*. *helveticus*, *W*. *confusa*	[40, 79]
		Tchoukoutou	Sorghum	*S*. *cerevisiae*, Lactic acid bacteria	[93]
		Iru	Locust beans	*B*. *subtilis*, *B*. *pumilus*, *B*. *licheniformis*, *Staphylococcus* spp.	[94]
	Burkina Faso	Dégué	Millet	*L*. *gasseri*, *L*. *fermentum*, *L*. *brevis*, *L*. *casei*, *Enterococcus* spp.	[52]
		Ben saalga	Millet	*L*. *plantarum* and other LAB	[84]
		Boule d’akassa	Millet	Unknown	[2]
		Gappal seche	Millet-milk	Unknown	[2]
		Bakalga	Kartade red sorrel (Hibiscus sabdariffa)	*Bacillus subtilis*	[95]
		Gari	Cassava	*L*. *plantarum*, *L*. *fallax*, *L*. *fermentum*, *W*. *paramesenteroides*, *L*. *brevis*, *Leuc*. *pseudomesenteroides (minor proportions)*, *Strep*. *lactis*, *Geotrichum*, *candidum*, *Corynebacterium manihot*	[84]
		Nono (milk curd)	Milk	LAB	[52]
		Wara	Milk	*Lactococcus lactis*, *Lactobacillus* spp.	[3]
	Senegal	Guedj	Fish	*L*. *lactis*	[96]
	Zimbabwe	Amasi	Cow Milk	*S*. *cerevisiae*, *C*. *colliculosa*, *C*. *lusitaniae*, *S*. *dairenensis*, *C*. *lipolytica*, *Dekera bruxillensis*, *C*. *tropicalis*, *Lc*. *lactis* subsp. *lactis*, *E*. *faecalis*, *Lc*. *lactis*, *Lb*. *casei*, *Lb*. *plantarum*, *Lb*. *paracasei*, *Lc*. *lactis* subsp. *lactis*, *Leuc*. *pseudomesenteroides*, *Lb*. *plantarum*, *Lb*. *delbrueckii* subsp. *lactis*, *Leuc*. *pseudomesenteroides*, *Leuc*. *mesenteroides* subsp. *dextranicum*, *Lb*. *helveticus*, *Lb*. *plantarum*	[78, 97]
	Sudan	Gariss	Camel Milk	*E*. *faecium*, *Lb*. *fermentum*, *Lb*. *helveticus*	[66, 98]
	Rwanda	Kiviguto	Cow Milk	*Leuc*. *mesenteroides* subsp. *mesenteroides*, *Lc*. *lactis*, *Leuc*. *Pseudomes enteroides*	[78]
	Zambia	Mabisi	Milk	*S*. *equinus*, *Citrobacter freundii*, *Acinetobacter ursingii*, *Lc*. *lactis*, *E*. *durans*, *Lb*. *brevis*, *Lb*. *kefiranofaciens*, *Leuc*. *garlicum*, *Lb*. *plantarum*, *Leuc*. *Pseudomesenteroides*, *S*. *thermophilus*	[78, 99]
Southern Africa	South Africa	Mahewu (magou)	Maize, sorghum, or millet	*L*. *delbrueckii* subsp. *bulgaricus*; *L*. *delbrueckii* subsp. *delbrueckii*; *Leuconostoc* spp., *heterofermentative lactobacilli*	[100]
		Sethemi	Milk	*Lactobacilli*, *lactococci*, yeast (*Debaromyces hansenii*, *Saccharomyces cerevisiae*, *Cryptococcus curvatus*)	[50]
		Kaffir beer (alcoholic beverage)	Kaffir com (or maize)	*Saccharomyces*, *Kluyveromyces*, *Candida*, *Pichia*, *Acetobacter*, *Lactococcus*, *Leuconostoc*, *Lactobacillus*, *L*. *kefiri*, *L*. *kefirgranum*, *Lactobacillus kefiranofaciens*, *L*. *delbrueckii*, *Lactobacillus parakefiri*, *L*. *acidophilus*, *Lactobacillus helveticus*, *Lactobacillus brevis*, *Lactobacillus paracasei*, *Lactobacillus casei*, *Lactobacillus fermentum*, *Lactobacillus gasseri*, *Lactobacillus plantarum*	[53] and [101, 102]
		Amasi	Cow milk	*S*. *cerevisiae*, *C*. *colliculosa*, *C*. *lusitaniae*, *S*. *dairenensis*, *C*. *lipolytica*, *Dekera bruxillensis*, *C*. *tropicalis*, *Lc*. *lactis* subsp. *lactis*, *E*. *faecalis*, *Lc*. *lactis*, *Lb*. *casei*, *Lb*. *plantarum*, *Lb*. *paracasei*, *Lc*. *lactis* subsp. *lactis*, *Leuc*. *pseudomesenteroides*, *Lb*. *plantarum*, *Lb*. *delbrueckii* subsp. *lactis*, *Leuc*. *pseudomesenteroides*, *Leuc*. *mesenteroides* subsp. *dextranicum*, *Lb*. *helveticus*, *Lb*. *plantarum*	[78]
		Mafi	Milk	*Leuc*. *mesenteroides* subsp. *dextranicum*, *Lb*. *delbrueckii* subsp. *lactis*, *Lc*. *lactis* subsp. *lactis*, *Lb*. *plantarum*	[72, 103]
	Botswana	Ting	Sorghum	*L*. *fermentum*, *L*. *plantarum*, *L*. *rhamnosus*	[52]
		Bogobe	Sorghum	Unknown	[52]
		Munkoyo	Maize	*Weisella* and *Lactobacillus * spp.	[99]
		Mabisi/Amasi	Milk	*Lactococcus*, *Lactobacillus*, and *Streptococcus* spp., and *Leuconostoc* spp.	[97]
		Amabere amaruranu	Milk	*Streptococcus thermophilus*, *Lactobacillus plantarum*, and *Leuconostoc mesenteroides*; *Yeasts*	[45]
		Marula/buganu	Amarula fruit	Yeasts	[47]
		Chibuku	Sorghum	*Lactobacillus* spp., *Saccharomyces cerevisiae*	[47]
		Madila	Cow or goat milk	*Lb*. *fermentum*, *Lb*. *plantarum*, *Lc*. *lactis*, *Lb*. *brevis*, *Lb*. *acidophilus*, *Lb*. *delbrueckii*	[66, 104]
		Umqombothi	Maize or sorghum	*Lactobacillus* spp., *S*. *cerevisiae*	[105]

Lb: *Lactobacillus*, Leuc: *Leuconostoc*, Lc: *Lactococcus*, W: *Weissella*, S: *Streptococcus*, E: *Enterococcus*, C: *Candida*, and ND: not determined

### Prokaryotes

The early stages of fermentation are dominated by lactic acid bacteria, following which yeasts increase and participate [13]. This is due to lactic acid bacteria relatively rapid growth rate. *Lactobacillus*, *Lactococcus*, *Leuconostoc,* and *Pedicoccus* are the four most common genera. *Saccharomyces cerevisiae* (eukaryotes), *Streptococcus,* and *Corynebacterium* are additional organisms (Table 1). *Penicillum*, *Aspergillus*, *Cladosporium,* and *Fusarium* are mold, which may also be present. In many traditional African fermentations, *L*. *plantarum* and *L*, *fermentum* strains dominate [14–16].

Diaz et al. [2] used 16S rRNA gene amplicon sequencing to examine the bacterial microflora of African fermented foods produced from various raw materials. In all of the samples, *Lactobacillus* was abundant, with the exception of those produced in laboratories. Fermentations of cereal, dairy, and cassava was dominated by genera in the order Lactobacillales. *Lactobacillus* and *Bacillus* were the most common genera in locust bean, while *Zymomonas*, *Bacillus*, and members of the order Lactobacillales were the most common in alcoholic beverages. This was the first observation of the genus *Zymomonas* in alcoholic fermentations [2].

Most studies describing on microbial communities of fermented foods from Africa agree that the dominant species are *L*. *plantarum* and *L*. *fermentum*. In some fermented cereal and dairy samples, other genera, like *Acetobacter*, are present in relatively high numbers. Also, numerous cereal and dairy samples contained potentially pathogenic genera such as *Clostridium* and *Escherichia* [17, 18].

*Lactobacillus*, *Streptococcus*, *Zymomonas*, *Leuconostoc*, and *Bacillus* are the most plentiful genera in alcoholic samples. *Lactobacillus* and *Leuconostoc* are the main groups present in palm wine [19, 20]. The process of fermenting locust bean is alkaline and typically involves *Lactobacillus* and other genera in the *Lactobacillales* order. *Bacillus* is responsible for fermenting the beans. Thus, high concentrations of this genus is expected and has been reported [21]. Bacteria like *B*. *subtilis*, *B*. *circulans*, and *B*. *megaterium*, which break down protein, are found in fermented condiments. Among these species, *Bacillus subtilis* is the most dominant and best suited for fermentation [22, 23]. According to Oguntoyinbo et al. [24], *B*. *subtilis* is the key factor affecting the production of mucilage, because of the high production of amylase, protease, and polyglutamic acid [24]. The fermentation of foods is also associated with microorganisms from other genera. *Pediococcus*, *Proteus*, *Escherichia*, *Micrococcus*, *Streptococcus*, *Staphylococcus*, *Pseudomonas*, *Alcaligenes*, *Enterococcus,* and *Corynebacterium* are some of the species that fall under this category [25, 26]. The bionetwork of fermented plant protein, particularly in the beginning of production, has been linked to members of the Enterobacteriaceae family [27, 28]. According to Ogueke and Aririatu (2004), the altered environment is probably the reason why these species do not flourished until the fermentation is complete [29]. As seen in African matured food sources, fermentation is initially started by various microorganisms, which eventually favors Gram-positive bacteria [12].

The phenotypic approach has been used frequently to identify the microorganisms in African fermented foods, but has inherent flaws and fails, to isolate and recognize non-cultivable microorganisms. Culture independent techniques such as amplicon sequencing can be used to study microorganisms of fermented food [30, 31]. However, not many studies have focussed on African food varieties [32, 33]. The latest research on the safety and processing methods of *ugba*, a fermented food condiment from Nigeria utilized both molecular and phenotypic approaches. The clone library technique identified new bacterial species of *Arthrobacter*, *Brevibacterium*, *Providencia*, *Empedobacter*, *Acinetobacter Elizabethkingia*, *Proteobacterium*, *Burkholderiales*, *Dysgomonas*, *Wautersiella*, *Flavobacterium,* and *Zymomonas*. These findings, subsequently, highlight the advantages of molecular techniques in the assessment of microbial flora. The microbial structure described for these products might be more complex than what is currently known.

### Yeasts

The role of yeasts in the fermentation of spontaneously fermented beverages and foods is crucial. Studies have shown that the diversity and succession of yeast species during fermentation are influenced by factors, such as commensal microorganisms, raw materials, hygiene, and processing techniques. Successions occur at species and strain levels according to intrinsic and extrinsic growth factors, which are constantly changing. Yeasts are necessary for flavor development and affect shelf life and nutritional value. Practical properties of yeasts in fermentation of food and beverages include fermentation of carbohydrates, formation of flavor compounds, stimulation of lactic acid bacteria, corruption of cyanogenic glycosides, development of proteins that debase tissues, the binding as well as degrading of mycotoxins, and probiotic properties[34, 35].

Many sub-Saharan African foods and beverages have been studied for the incidence of yeasts. Yeasts are vital in the processing of African fermented food and beverages, and the related data are exceptionally disorganized. This makes it difficult to outline their incidence, identity, effects, and interactions. The current review assembles current information on yeasts in African fermented food and beverages, concentrating on the species and strain.

*Saccharomyces cerevisiae* is the predominant yeast isolated from the majority of fermented products, followed by *Pichia kudriavzevii*, *Candida tropicalis,* and *Kluyveromyces marxianus* [36]. *S*. *cerevisiae* dominated the fermentation of solid foods *kenkey* and *mawè*, as well as *ogi* (non/low-alcoholic drink) [37]. *S*. *cerevisiae* was surmised to be the primary fermenting agent for 93% of African alcoholic drinks, which are mostly derived from cereal crops. Other types of alcoholic drinks, such as palm wine made from sap [19, 38], fermented dairy products like *Nunu* [39, 40], *rob,* and *suusac* [41] also had *S*. *cerevisiae* as the predominant species.

Low concentrations of *S*. *cerevisiae* have been recorded in indigenous African fermented products such as fufu [42], lafun [15], and adjuevan, non/low-alcoholic drinks such as bushera [43] and togwa [44], and some of the fermented dairy products such as *amabere amaruranu* [45], *kefir* [46], *amasi* [47], *nyarmie* [48], *mashita* [49],and *sethemi* [50].

### Probiotic Properties of Strains

Live microorganisms are administered to improve the health of the host. These microorganisms are commonly referred to as probiotics. The genera *Lactobacillus*, *Bifidobacterium*, *Lactococcus*, *Enterococcus*, *Leuconostoc,* and *Streptococcus* comprise the probiotic lactic acid bacteria. Yogurt, fermented milk, and fermented foods are all sources of probiotics [106]. Several potentially nutritious and health-promoting components (mainly microorganisms) are found in fermented African foods. However, despite the growing interest in lactic acid bacteria as a probiotic, available data on novel applications as a probiotic are rare, particularly from Africa. Only a few probiotic products have made it past clinical trials due to stringent international regulations. There is a lack of data on the probiotics market share in Africa compared to the rest of the world. In South Africa, the market for fortified foods (especially baby cereals), supplements, and fermented dairy products appears to be fairly established.

It is expected that lactic acid bacteria members, including those found in fermented condiments such as *Iru*, *Ugba*, and *Ogiri*, and fermented milk products such as *Nunu* and yoghurt *Wara* have been the focus of research from a microbiological perspective. These include *Lactobacillus*, *Lactococcus*, *Pediococcus*, and *Weissella* species, with particular emphasis on the presence of the last two in fermented cereal-based foods such as *Ogi* [107, 108]. Table 2 summarizes research into the probiotic potential of strains found in traditional African food products. The term "potentially" is used when indications of health benefits do not meet the necessary criteria for the usage of the term "probiotic." Before the term "probiotic" could be used to describe these microorganisms, additional research, including clinical trials, is required.

**Table 2:** Probiotic properties of the strains isolated from African fermented foods

Product	Microorganisms tested for probiotic potential	Probiotic properties	References
*Nono*	*Enterococcus faecium* strains	• Tolerant to bile salts • Possess antagonistic activity against foodborne pathogens including *Bacillus cereus* and *Listeria monocytogenes* • Strong acidification properties	[113]
Maize dough	*L*. *plantarum* *L*. *fermentum*	•Poorly adherent to Caco-2 • Survived 4 h at pH 2.5 • Antimicrobial activity toward *L*, *monocytogenes*, *S*, *aureus* and *B*, *cereus strains* • Products not tested in humans	[114]
*Fufu* and *ogi*	*Pediococcus* *L*. *plantarum*	• Produced H_2_O_2_ • Tolerant toward bile and low pH • Good adherence capacity to mucus-secreting HT29 MTX epithelial cells	[92]
Pearl millet slurries	*L*. *fermentum*	• Containing genes associated with survival during passage through the gastrointestinal tract • Bile salt tolerance • Folate and riboflavin synthesis	[115]
Kimere	*L*. *fermentum*	• Survived medium containing 3% oxgall • Survived incubation at pH 3 for 3 h	[116]
*Kule-naoto*	*Lactobacillus paracasei*, *L*. *plantarum*, *L*. rhamnosus, isolates *of L*. *acidophilus*	• Resistance to gastric juice and bile, while some expressed bile salt hydrolase activity •Assimilated cholesterol • Adhesion to HT29 MTX cells • High survival rate under simulated stomach acidic conditions • Adhered well to enterocytes and prevented invasion of pathogens in cell culture • Expressed antimicrobial activity toward foodborne pathogens and stimulated IL-8 production in intestinal epithelial cells in vitro	[117]

Lei and Jacobsen investigated the main lactic acid bacteria present in *koko* and koko sour water from different locations in Ghana, with regard to their genotype, fermentation patterns, tolerance to low pH and bile salts, and antimicrobial properties [109]. The presence of oxgall bile had no effect on the growth of any of the lactic acid bacteria isolates. In pH 2.5 media, the strains survived, but were unable to grow. The strains were tolerant to acid and bile with antimicrobial activity in fresh *koko* sour water at levels of 10^8^ colony-forming units/mL. Pinto et al. investigated lactic acid bacteria in African fermented millet, including diversity, naming, and as a possible as a probiotic for the local community. Additionally, the probiotic properties of *Lactobacillus* strains and human intestinal isolates from traditional African fermented milk products were examined. The researchers found that the survival rate of the strains was influenced by factors, such as acidic conditions (pH 2.5), pepsin, lysozyme, and milk. Under simulated gastrointestinal conditions, five strains were identified as *L*. *plantarum* and two as *L*. *johnsonii* survived well. Antimicrobial activity was also demonstrated by these strains, probably because of the production of organic acids. Bile salt hydrolase activity was present in all strains, but β-galactosidase activity was only present in *L*. *plantarum* strains. An investigation into how various dietary treatments affect the permeability of the intestinal wall during acute diarrhea was performed on 87 hospitalized children aged 6 to 25 months in Tanzania. According to [110], fermented porridge performed better than conventional porridge or porridge digested with amylase in the treatment of intestinal permeability.

Mensah et al. (1995) included mothers from Ghana and Nigeria to compare fermented and non-fermented maize–soybean porridges with conventionally fermented maize-only porridges [111]. According to their findings, infants who consumed fermented porridges had significantly higher daily nutrient intakes than those who ate regular porridge. In many areas of Nigeria, *Ogi* liquor, which is water made from fermented cereal pulp, is given to infants by nursing mothers, to cure their illness. Adebolu et al. found that the *ogi* liquor, which included *Lactobacillus* species among others, inhibited pathogens (common diarrhoeal bacteria) in southwest Nigeria. Zakpaa et al. reported that Ghanaian fermented meats have lactic acid bacteria species, *Staphylococcus* spp., *Streptococcus* spp., and *Micrococcus* spp. in their microbial flora [112].

The species of *Staphylococcus* are identified as pathogenic, along with *Streptococcus* spp. and lactic acid bacteria species as probiotics. Based on these findings, robust probiotic starter culture to improve the quality of fermented meats by preventing the growth of potentially pathogenic organisms could be beneficial.

Since the term "probiotic" may be unfamiliar to a number of African communities, any mention of such products must coincide with an educational campaign. Few studies have been conducted to determine whether microorganism in African fermented foods meets the requirements to be termed probiotics. As a result, it is necessary to train people and develop technologies to improve the production processes, starting with accurate strain-level characterization of probiotic organisms found in fermented foods.

## Role of Microorganisms in Improving Food Security in Africa

Although Africa has a vast amount of uncultivated and fertile land, it continues to face food security challenges despite the potential to feed its growing population and more. Approximately 240 million individuals in sub-Saharan Africa do not have access to enough food to maintain an active and healthy lifestyle. [118]. One possible approach to tackle this problem involves utilizing microorganisms in different ways to maintain food quality, enhance its nutritional value, increase food output, or even provide food as a resource.

Fermented foods have been subjected to enzymatic actions of microorganisms [119]. Fermentation is a centuries-old way of preserving surplus vegetables, fruit, meats, and grains and can enhance the overall flavor of the meal. Fermentation produces acids that avert the growth of spoilage-causing pathogens, making food safer and increasing its shelf life, especially when refrigeration or other forms of food processing are unavailable. Additionally, fermentation enhances the food's sensory properties, improves its nutritional value, and may also improve digestibility [120, 121].

Limiting the expansion of destructive microorganisms can lower the chances of pathogenic diarrhea, which is a primary reason for the death of infants in sub-Saharan Africa. Raw sorghum flour has more than 2,400 colony-forming units per gram (cfu/g) of *Escherichia coli*, whereas the count is notably lower in fermented dough [102]. Moreover, the fermented dough does not contain any *Salmonella* spp., which were present in different sorghum varieties. [122].

Several types of fermented food are commonly consumed in Nigeria, including *gari* and *fufu*, which are two popular types of fermented cassava. Other examples of fermented products *ogi* (maize), *dawadawa* (African locust beans), *ogiri* (castor oil seeds), *ugba* (African oil beans), *kunu zaki* (maize drink), *shekete* (palm wine), and traditionally fermented milk and cheese. The microorganisms responsible for most of these fermentations are yeasts and lactic acid bacteria [123].

Microorganisms are used to improve food security globally and can play a significant role in food security in Africa. Therefore, to increase the effectiveness of food production systems and counteract the negative impacts of agricultural production, including meat production, innovative technologies are crucial. While the practice of utilizing microorganisms to ferment food has been around for centuries, the concept of using microorganisms as a source of food has only gained widespread acceptance in recent times [124]. The utilization of microbial biomass as a food or feed source is known as "microbial protein" or MP. Around 75% of the dry biomass of MP comprised protein, including all the essential amino acids. In addition, MP is a good source of vitamins, minerals, and other nutrients [125].

**Fig. 1:**
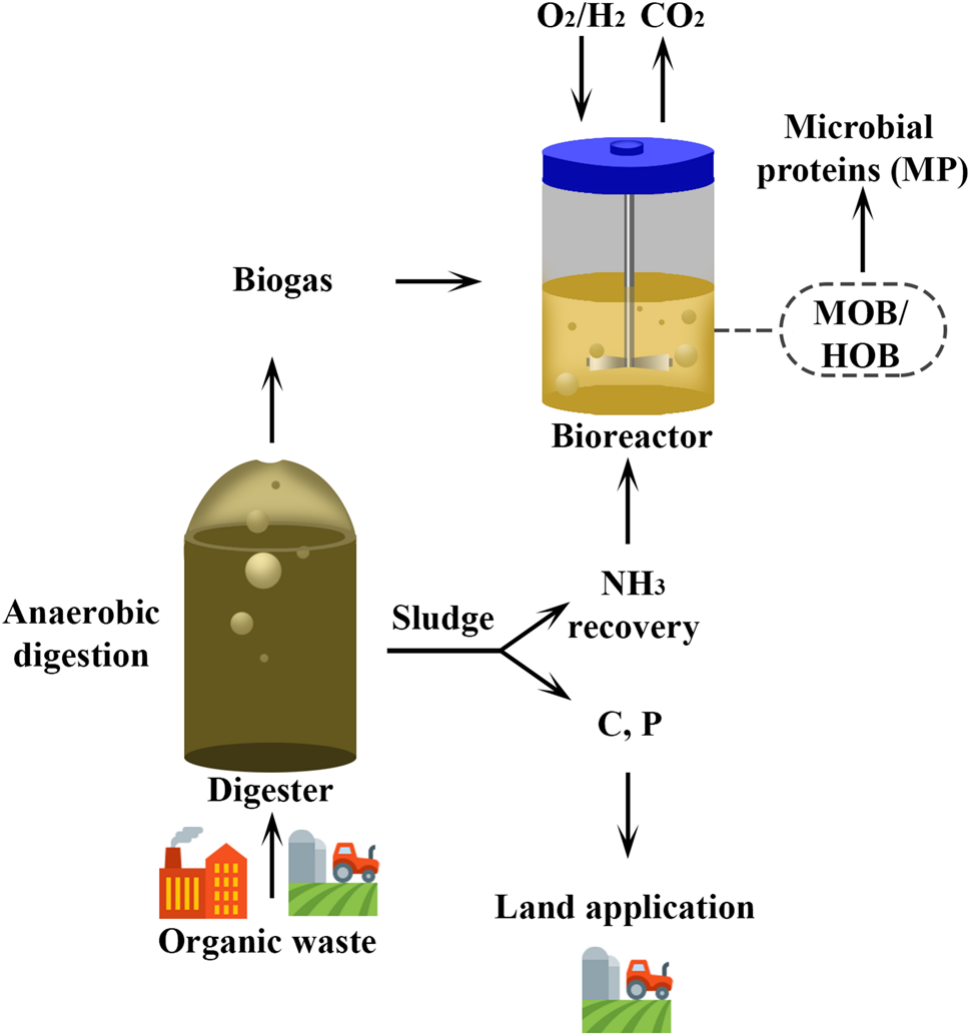
Schematic representation of microbial protein (MP) production. Organic and other carbon rich waste is anaerobically digested by acidogenic/methanogenic bacteria in an anaerobic digester. The released biogas (CH_4_ and CO_2_) along with NH_3_ from the digestate sludge is injected into the bioreactor. The introduced O_2_ and H_2_ are used by methane-oxidizing bacteria (MOB) and hydrogen-oxidizing bacteria (HOB), respectively, which collectively convert organic wastes, and agricultural by-products into microbial proteins (MP). The MP can be used as food additives and the C and P from the sludge can be used for land application (biofertilizers). The figure illustration has been accomplished with Adobe Photoshop CS6 64 bit

Bioreactors, depicted in Fig. 1, are closed systems that can generate MP. The design and operation of these systems vary to create ideal conditions based on the organism's characteristics, such as being a phototroph or chemotroph, as well as an autotroph or heterotroph. Bioreactors are much more effective than growing crops in open fields or keeping livestock, because the growth parameters can be kept stable, nutrients are used with near 100% efficiency (nutrients can be added to match demand precisely), the footprint for water and land use is small, and there is no need for pesticides, antibiotics or vaccines [124]. Examples of methane-oxidizing bacteria are *Methylococcus capsulatus* and *Methylomonas methanica* [126]. Examples of hydrogen-oxidizing bacteria are *Alcaligenes eutrophus*, *Seliberia carboxydohydrogena,* and *Ralstonia eutropha* [127]. Microfungi, notably *Fusarium venenatum*, and microalgae have also been used.

Another advantage of microbial protein is that it can be produced in a variety of forms such as powders, flakes, or bars. This makes it versatile and easy to use in a wide range of food products, from traditional dishes to modern food products such as protein bars, protein shakes, and meat alternatives.

### Usefulness of Microbial Products (Metabolites)

Food-processing technologies modify raw materials to obtain products with desirable quality characteristics, which meet nutritional and functional needs. The process of food fermentation involves the use of microbial growth technology to transform raw food materials into a variety of value-added products on various substrates. [79, 102]. This is one of the traditional methods of food processing and preservation in Africa, where there is a rich history of fermented foods. Africa is believed to be the origin of humankind, and its long-standing food-processing techniques, including fermentation, have been passed down through generations for centuries. Additionally, there are both modern and basic food-processing technologies, with simple techniques such as drying, salting, smoking, and fermentation [118, 128, 129].

Traditional fermented dairy products that have been consumed for centuries. These products continue to be important sources of essential nutrients and income for local populations [72, 130]. The type of fermented dairy products varies in different African countries. The fermented foods produced in African countries include non-alcoholic and alcoholic beverages like wine and *pito*. Fermented dairy products also include yogurt, yogurt-like foods, and beverages [131]. These fermented foods are made from animal sources such as cow milk, camel milk, buffalo milk, goats’ milk, ewe milk, meat, and fish as well as plant-based raw materials such as starchy vegetable proteins, root crops, fruits, and leaves [132, 133].

Milk has had a substantial economic and nutritional impact in African countries, predominantly those that rely on cattle-keeping. These regions comprise North Africa, the Sudanian Savanna (Burkina Faso, Gambia, Senegal, and Southern Mali), as well as northern countries such as Ghana, Côte d'Ivoire, Togo, Benin, Guinea, and Nigeria, and the highlands of East Africa [134]. Generally, African fermented dairy products are enriched in nutrients, micronutrients, delivering high-quality proteins, vitamins, and energy-containing fats [135, 136], but notably vitamins. The eight vitamins in African beers, for instance, are well documented. African fermented dairy products can be categorized into three functional groups, staples, beverages, and the relishes and sauces [132, 137].

The production of African fermented dairy products involves two methods. The first is spontaneous fermentation, which occurs at household level and produces yogurt-like products [138, 139]. These products have a diverse microbial community consisting of lactic acid bacteria and yeasts and are rich in nutrients. African fermented dairy products also provide benefits such as inhibiting harmful microorganisms and improving digestion and nutritional value. Second, a few products as *Madila* in Botswana and *Amasi* in South Africa are produced commercially [131, 140]. A list of fermented milk products is shown in Table 1. These fermented milk products such as *Amabere*, *Amasi*, *Ergo*, *Fene*, *Gariss*, *Ititu*, *Kefir*, *Kindirmou*, *Kiviguto*, *Kule-naoto*, *Kwerionik*, *Leben* (*lben*), *Mabisi*, *Madila*, *Mafi*, *Makamo*, *Masse*, *Mursik*, *Nunu*, *Nyarmie*, *Omashikawa*, *Pendidaam*, *Rob*, *Suusac*, and *Zabady* are made from good quality raw milk [130]. A significant aspect of African pastoral societies is the importance of milk, not only as a source of sustenance but also plays a crucial role in their social, economic, and cultural practices. Milk plays a vital role in the organization of society, trade, eating habits, technological advancements, and cultural heritage. According to Mattiello and colleagues [72], dairy products are classified into five groups based on production method and type, fresh cheese, ripened cheese, fermented milk, butter, and dairy by-products [72].

The significance of fermented milk in Africa should be highlighted. It plays a crucial socio-economic role and is widely used [91]. There are several traditional fermented milk products in Africa (Table 1). The production methods for these products vary depending on the local microbiota, which is influenced by the climate of each area. The consumption of traditional fermented milk products has health benefits, such as potential probiotic properties of some lactic acid bacteria. Probiotics refer to live microorganisms that offer health advantages to the host if consumed in proper amounts [141]. The presence of live bacteria in fermented milk can impede the growth and spread of harmful microorganisms responsible for causing food-related illnesses such as diarrhea [142, 143]. *L*. *acidophilus*, *L*. *bulgaricus*, *B*. *bifidum,* and *S*. *thermophilus* are among the lactic acid bacteria that impede colonizing pathogens in the gastrointestinal tract [143].

### Microbial Biomarkers for Assessing Food Quality

Biomarkers, including proteins and metabolites, play a crucial role in assessing the quality of food. By measuring specific molecules present in food samples, scientists and food industry professionals can gain valuable insights into its safety, nutritional content, and overall quality. Therefore, exploring the significance of biomarkers in food quality assessment and highlighting their potential applications are essentially important [144]. They provide objective measurements that can help in determining the freshness, nutritional value, and potential hazards associated with food products. One of the key implications of biomarkers is in the food industry for determining the freshness of perishable products. As food undergoes spoilage, certain biomolecules undergo changes that can be detected and measured. For example, the degradation of proteins or the production of volatile compounds can indicate the presence of spoilage bacteria. By monitoring these biomarkers, food manufacturers and distributors can assess the quality of their products and make informed decisions regarding their shelf life [145]. Analyzing biomarkers data requires a systematic and rigorous approach. From data quality control and preprocessing to statistical analysis and interpretation, each step plays a crucial role in extracting meaningful insights from the biomarker data. By following these steps, food scientists can make informed decisions regarding food safety, quality control, and regulatory compliance [146, 147].

### Protein Biomarkers in Food Quality Assessment

Proteins are fundamental components of food and essential macromolecules found in all living organisms. They are not only indicators of nutritional content but also play a vital role in determining the authenticity and safety of food products. By analyzing the presence, quantity, or modifications of specific proteins, researchers can detect adulteration, contamination, or the presence of allergens in food. Specific protein biomarkers can indicate the presence of allergens, pathogens, or spoilage in food products. For instance, the detection of allergenic proteins in processed foods helps in identifying the potential health risks for individuals with allergies. Similarly, specific pathogen-related proteins can indicate contamination, aiding in rapid response and preventing foodborne illnesses. Similarly, the presence of gluten proteins can help in identifying products that are gluten-free, ensuring the safety of consumers with gluten intolerance [148, 149].

### Metabolite Biomarkers in Food Quality Assessment

Metabolites, small molecules produced during metabolic processes, are also involved in various biochemical processes within living organisms. They can serve as valuable biomarkers for assessing food quality due to their sensitivity to changes caused by processing, storage, or contamination. By analyzing the levels and profiles of specific metabolites, scientists can evaluate the freshness, nutritional value, and potential hazards of food products [150]. In brief, various analytical techniques are employed to analyze the levels and profiles of specific metabolites in food. These techniques can be broadly categorized into targeted and untargeted metabolomics approaches. Targeted metabolomics focuses on the quantification of specific known metabolites. This approach utilizes analytical techniques such as gas chromatography (GC), liquid chromatography (LC), or mass spectrometry (MS) to identify and quantify the predetermined metabolites. Targeted metabolomics offers high sensitivity, specificity, and reproducibility, making it suitable for analyzing specific metabolites of interest. Untargeted metabolomics involves the comprehensive analysis of all detectable metabolites present in a sample. This approach utilizes advanced analytical techniques, such as nuclear magnetic resonance (NMR) spectroscopy and high-resolution mass spectrometry (HRMS), to identify and quantify various metabolites. Untargeted metabolomics allows for a holistic understanding of food metabolite composition, enabling the discovery of novel metabolites and metabolic pathways [151]. After obtaining the analytical data, the analysis of metabolites in food involves data processing, statistical analysis, and interpretation. Advanced computational tools and software are employed to process the vast amount of data generated by analytical techniques. Statistical analysis helps to identify the significant differences in metabolite levels between samples and provides insights into the relationships and interactions between metabolites. The interpretation of the data allows researchers to draw meaningful conclusions about the metabolite profiles and their potential implications for food quality, nutritional value, and health benefits [152].

Furthermore, they can provide insights into food products' nutritional content, flavor, and freshness. Metabolite profiling can help identify the changes in food composition due to storage conditions, processing methods, or adulteration. For example, the analysis of volatile metabolites can reveal the degradation of fats and oils, indicating rancidity and the potential loss of nutritional value. Besides, the presence of specific metabolites can indicate the presence of microbial contamination (determining the presence or absent of their metabolites(, helping to prevent foodborne illnesses [153].

### Other Biomarkers

Apart from proteins and metabolites, various other biomarkers, such as DNA, RNA, enzymes, and volatile compounds, can be utilized to assess the quality of food. DNA-based biomarkers are specific DNA sequences that can distinguish between different species or origins. These biomarkers are unique to each organism and can be used to determine the presence or absence of a particular species in a given food product. DNA-based biomarkers, such as mitochondrial DNA (mtDNA), nuclear DNA (nDNA), and single-nucleotide polymorphisms (SNPs), can be used to identify the species or origin of food products, ensuring their authenticity [154]. On the other hand, RNA biomarkers can provide insights into gene expression changes during food processing or storage, help in monitoring its quality, while enzymes can be used to assess the freshness of food products by measuring their activity levels. Additionally, volatile compounds can serve as indicators of flavor, aroma, and spoilage, enabling sensory quality evaluation [155].

### Applications of Biomarkers in Food Quality Assessment

- Shelf life determination: Biomarkers, such as volatile organic compounds (VOCs), enzymes, proteins, and DNA fragments, can assist in predicting the shelf life of food products. By monitoring the changes in these biomarkers, manufacturers can gain valuable insights into the degradation and spoilage processes of their products, establish appropriate storage conditions, and ensure consumer safety.
- Quality control: Analysis of biomarkers allow to detect contaminants, adulterants, and other quality-related issues in food, facilitating effective quality control measures.
- Nutritional profiling: Biomarkers can provide insights into the nutritional composition of food, inform consumer to make their specific choices about their diet.
- Authentication and traceability: Biomarkers can be used to verify the origin, authenticity, and traceability of food products, ensuring adherence to label regulations [146, 156].

The use of biomarkers, such as proteins, metabolites, and other molecular indicators, have revolutionized the assessment of food quality. Their measurement and analysis enable food scientists and industry professionals to make suitable decisions regarding safety, composition, nutritional content, the authenticity of food products, and overall quality of food products. Additionally, this assessment also aid in the development of quality control measures [144, 157]. By analyzing the presence, quantity, or modifications of these biomarkers, scientists and food regulators can ensure the production of safe and nutritious food for consumers. Biomarker analysis techniques continue to improve, which has tremendous potential for improving food quality evaluation. On the other hand, by leveraging biomarker-based approaches, the food industry can enhance consumer trust, ensure regulatory compliance, and improve overall food safety and quality [157].

## Future Perspectives and Opportunities

In future studies on the microbial diversity of African food and drinks, the identification and characterization of microorganisms found in traditional fermented products should be prioritized, and their effects on food safety and nutrition were evaluated. Research should examine the potential of microorganisms to produce fermented food and new food products. Additionally, exploring methods to improve storage and preservation of food and ways to enhance their nutritional content would be beneficial. One potential research area is exploring the health advantages in consuming fermented food and drink, and how these benefits might differ depending on the population and region. Therefore, it is crucial to investigate how alterations in food processing, production, and distribution methods might affect the safety and variety of fermented foods and beverages in Africa. There is a significant chance to enhance food security by encouraging the utilization of biofertilizers and boosting the creation and consumption of microbial protein.

## Conclusion

Understanding the microbial diversity of African foods and beverages is essential for promoting food security and human health, particularly in Africa. This is due to the numerous benefits that these microorganisms provide, ranging from probiotic properties that improve human health to metabolites that have potential in the food industry. African fermented foods are made from a variety of raw materials and provide many health benefits. Knowledge of the microbial ecology of natural fermentations can identify the biomarkers to evaluate the quality of fermented foods and optimal starter cultures for food production. With more research into the microbial diversity of African foods and beverages, food security and human health can be improved. Additionally, microbial proteins are useful in the food industry and future research should continue to explore their role as a food source to improve in food security.
